# Chenodeoxycholic Acid Has Non-Thermogenic, Mitodynamic Anti-Obesity Effects in an In Vitro CRISPR/Cas9 Model of Bile Acid Receptor TGR5 Knockdown

**DOI:** 10.3390/ijms222111738

**Published:** 2021-10-29

**Authors:** João S. Teodoro, Ivo F. Machado, Ana C. Castela, João A. Amorim, Ivana Jarak, Rui A. Carvalho, Carlos M. Palmeira, Anabela P. Rolo

**Affiliations:** 1 Department of Life Sciences, University of Coimbra, Calçada Martim de Freitas, 3000 Coimbra, Portugal; imachado@cnc.uc.pt (I.F.M.); catycastela@hotmail.com (A.C.C.); rac@uc.pt (R.A.C.); palmeira@ci.uc.pt (C.M.P.); anpiro@ci.uc.pt (A.P.R.); 2 Center for Neurosciences and Cell Biology, Rua Larga, Faculdade de Medicina, University of Coimbra, 3000 Coimbra, Portugal; joao_amorim@hms.harvard.edu; 3 IIIUC–Institute of Interdisciplinary Research, University of Coimbra, Pólo II da Universidade de Coimbra, 3000 Coimbra, Portugal; 4 Department of Genetics, Blavatnik Institute, Paul F. Glenn Center for the Biology of Aging, Harvard Medical School, Boston, MA 02115, USA; 5 Department of Pharmaceutical Technology, Faculty of Pharmacy of the University of Coimbra, Azinhaga de Santa Comba, Pólo das Ciências da Saúde, 3000 Coimbra, Portugal; jarak.ivana@gmail.com

**Keywords:** CDCA, TGR5, mitochondria, mitophagy, CRISPR/Cas9, 3T3-L1

## Abstract

Bile acids (BA) have shown promising effects in animal models of obesity. However, the said effects are thought to rely on a thermogenic effect, which is questionably present in humans. A previous work has shown that the BA chenodeoxycholic acid (CDCA) can revert obesity and accelerate metabolism in animal and cell culture models. Thus, the aim of this study was to understand if this obesity reduction is indeed thermogenically-dependent. A CRISPR/Cas9 model of TGR5 (BA receptor) knockdown in 3T3-L1 adipocytes was developed to diminish thermogenic effects. Various parameters were assessed, including mitochondrial bioenergetics by Seahorse flux analysis, oxidative stress and membrane potential by fluorometry, intermediary metabolism by NMR, protein content assessment by Western Blot, gene expression by qPCR, and confocal microscopy evaluation of mitophagy. CDCA was still capable, for the most part, of reversing the harmful effects of cellular obesity, elevating mitophagy and leading to the reduction of harmed mitochondria within the cells, boosting mitochondrial activity, and thus energy consumption. In summary, CDCA has a non-thermogenic, obesity reducing capacity that hinges on a healthy mitochondrial population, explaining at least some of these effects and opening avenues of human treatment for metabolic diseases.

## 1. Introduction

Obesity is a worldwide epidemic, recognized by the World Health Organization as an established problem for both developed and developing nations for the burdens exerted on national healthcare systems due to the massive drain of resources resulting from associated pathologies [1]. The major issue lies with associated pathologies to obesity, ranging from diabetes, cardiovascular problems, and to even certain types of cancer [2]. Despite extensive research and attempts to find a cure for obesity, pharmacological intervention has failed to resolve most of obesity’s issues, where only typically a surgical bariatric reduction intervention associated with a dramatic lifestyle alteration has shown any consistent gains.

In the past couple of decades, research on the anti-obesity effects of bile acids (BA), in particular cholic and deoxycholic acids (CA and CDCA, respectively) has shown tremendous potential to achieve this goal of a single pharmacological cure for obesity. Studies in obese mice [3,4,5,6] have demonstrated that BA can reverse obesity and ameliorate associated conditions. Initially, most studies attributed these effects to the activation of an orphan nuclear BA receptor, the Farnesoid X Receptor (FXR) [5,7,8]. However, later studies have questioned the role of FXR in metabolic improvement, for it appears that specific FXR agonism could in fact be detrimental [6,9]. As such, the consensus for BA effects now hinges on the activation of another BA receptor, the Takeda G-protein receptor 5, or TGR5 [6]. TGR5 activation is reported to lead to the intracellular brown adipose tissue (BAT) generation of triiodothyronine (T3) from internalized thyroxine (T4). T3 is a known thermogenic hormone, leading to the activation of a non-shivering thermogenic program in BAT, which is responsible for the controlled dissipation of mitochondrial membrane potential (ΔΨ) as heat (due to the activation of the BAT-specific mitochondrial uncoupling protein 1-UCP1, also known as thermogenin), resulting in the burning of excess nutrients and thus leading to decreased obesity [10,11]. TGR5 activation by CDCA was even shown in humans, albeit non-obese ones [12]. This is particularly interesting since adult humans do not possess large depots of BAT as small mammals and infants, which heavily rely on BAT for the maintenance of the core temperature, while larger mammals typically hinge on the skeletal muscle spontaneous contraction (also known as shivering) for temperature maintenance needs. As such, it would appear that the use of BA for tackling human obesity reached a dead-end due to the dependency of these effects on BAT activity. In fact, a pair of recent publications highlighted how hypothalamic TGR5 is required, in a mice model of obesity, for dietary BA effects [13,14].

We have, in a previous work [3], questioned the established TGR5 model, where it was shown that exposing a cell line of cultured white adipocytes (3T3-L1 adipocytes) to CDCA also significantly reduced adiposity, with a concomitant increase in metabolic fluxes and mitochondrial activity, without the input of thyroid hormones. This work suggested the possibility of BA having other, non-TGR5-dependent, effects on metabolism. Here, we aimed to understand if these effects were truly independent of TGR5. To achieve this, a CRISPR/Cas9 3T3-L1 model of TGR5 knockdown was developed and we tested if CDCA could still show metabolic acceleration and obesity reduction. This work reports that CDCA could indeed reduce the obesity of these cells, increase their metabolic rate, and improve overall metabolism as before, but with limited TGR5 involvement. This work proposes that the low dose of CDCA used in these studies (50 µM) is enough to cause a non UCP1-dependent thermogenic dissipation of ΔΨ due to its known protonophoric effect [15], while not harmful to the cells, as previously shown [3], thus concluding that BA still have an enormous potential for human use, despite the absence of large depots of UCP1-carrying BAT.

## 2. Results

### 2.1. Confirmation of TGR5 Ablation Using CRISPR/Cas9 in 3T3-L1 Adipocytes

**Figure 1:**
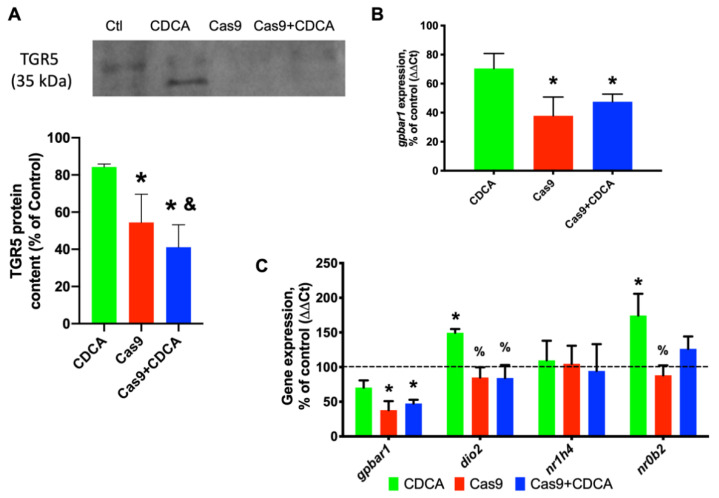
Confirmation of the experimental model. (**A**) Western Blot representative image for TGR5 and quantification of protein content; (**B**) Gene expression (as percentage of control) for *gpbar1*, TGR5′s gene; and (**C**) Gene expression (as percentage of control) for the TGR5 downstream effector deiodinase 2 (D2, *dio2*), and the other BA receptor FXR (*nr1h4*) and its downstream gene, the small heterodimer protein (SHP, *nr0b2*). Data are derived from six independent experiments, and bars represent means ± SEM. * indicates a statistically significant difference vs. Control (*p* < 0.05); % indicates a statistically significant difference vs. CDCA (*p* < 0.05); & indicates a statistically significant difference vs. Cas9 (*p* < 0.05).

In order to confirm that the Cas9 activation and thus TGR5 removal was efficient, both real-time PCR and Western Blot analysis were performed. As can be seen in Figure 1, Cas9 activity led to a decrease in *gpbar1* (TGR5 gene) expression of approximately 70% and a protein content reduction of roughly 50%. Furthermore, no effect was found in the other BA signaling pathway, i.e., involving FXR. As such, all of the experimental protocols that follow were performed with this method, i.e., achieve differentiation after 14 days, activate Cas9 for 4 days, and expose cells to CDCA (keeping Cas9 activated) for another 96 h.

### 2.2. The Metabolic Characterization of TGR5 Knockdown Adipocytes Shows an Accelerated Metabolism

To better understand the previously reported effects of CDCA on 3T3-L1 adipocytes [3], different metabolic parameters were analyzed in these cells. First, reporting was done about triglyceride (TG) deposition in these cells by Oil-Red O staining. As can be seen in Figure S1A, the TG content was efficiently decreased by CDCA in 3T3-L1 adipocytes by roughly 20% (as previously reported, [3]), independently of the TGR5 presence.

The physiological impact of CDCA on cellular metabolism was estimated by the NMR-based metabolomics approach. Changes in the total metabolome of aqueous extracts were evaluated by pair-wise comparisons of experimental groups (Figure S1B). Although several groups of polar metabolites have been identified, including amino acids, organic acids, and sugars, changes (VIP > 1) were only observed in acetate, lactate, and myo-inositol levels. PLS-DA analysis demonstrates that the separation between the Control and CDCA groups is caused by the increased levels of [U-^12^C]acetate in the Control group and this was confirmed by the univariate analysis. Increased [U-^12^C]acetate also distinguishes between the Control and Cas9 groups. Although a tendency towards the increase in [U-^13^C]acetate and [U-^12^C]lactate in the Control group is also suggested by PLS-DA VIPs, it does not show statistical significance in univariate analysis. Similarly, analysis of VIPs and univariated analysis indicated an increase of [U-^13^C]lactate in the Cas9 group when compared to the CDCA + Cas9 group. [U-^13^C]lactate is also increased in the Cas9 group. No significant changes were found in the myo-inositol content between the experimental groups.

The analysis of cell culture media by ^1^H NMR (Figure S1C) and of aqueous cell extracts by ^13^C NMR (Figure S1D) allowed for an evaluation of the effects of CDCA in cellular metabolism. The fractional enrichment of acetate in cell culture media was significantly higher in CDCA (0.27 ± 0.03) and CDCA + Cas9 (0.30 ± 0.04) treated cells than in Control (0.020 ± 0.02) and Cas9 (0.019 ± 0.03) groups. This higher enrichment of the acetate pool is consistent with a higher contribution of OXPHOS relative to glycolysis in the presence of CDCA as previously reported [3]. Moreover, the levels (in mM/mg protein) of [U-^13^C]lactate were significantly higher in the Control (3.95 ± 0.33) and Cas9 (3.84 ± 0.28) groups than in CDCA (2.84 ± 0.22) and CDCA + Cas9 (2.54 ± 0.31) cells, indicative of a higher contribution of glycolysis in both Control and Cas9 groups.

From the analysis of the glutamate C4 (Glu-CA) multiplet pattern (expansions in Figure S1D), the quartet multiplet (Q) component was significantly higher in the CDCA (0.48 ± 0.04) and CDCA + Cas9 (0.52 ± 0.03) cells than in the Control (0.27 ± 0.04) and Cas9 (0.030 ± 0.05) cells. The higher the Q multiplet component, the faster the TCA cycle turnover, suggesting a higher contribution of [U-^13^C] glucose and *de novo* synthesized fatty acids to OXPHOS [3]. In short, the NMR data indicate a higher metabolic turnover rate in CDCA-exposed groups, independently of TGR5 knockdown.

### 2.3. The Mitochondrial Activity Profile of CDCA-Exposed Adipocytes Is Unaltered by TGR5-Knockdown

To better understand the data from the last section, mitochondrial activity was assessed with resource to a variety of assays. The mitochondrial membrane potential (ΔΨ) was quantified by TMRM fluorescence and reactive oxygen species generation was evaluated by H_2_DCF-DA fluorescence, and both were measured in a Victor3 spectrophotometer. Figure S2A clearly shows that CDCA is capable of decreasing mitochondrial ΔΨ, an effect that is not lost when TGR5 is knocked down. Similarly, the knockdown of TGR5 did alter oxidative stress levels as measured by DCF fluorescence, with CDCA effectively reducing oxidative agents’ levels, namely H_2_O_2_. These data sets appear to point to a protonophoric effect of this BA, which could explain why CDCA is capable of reducing intracellular energy reserves, in this in vitro, BAT-removed model.

**Figure 2:**
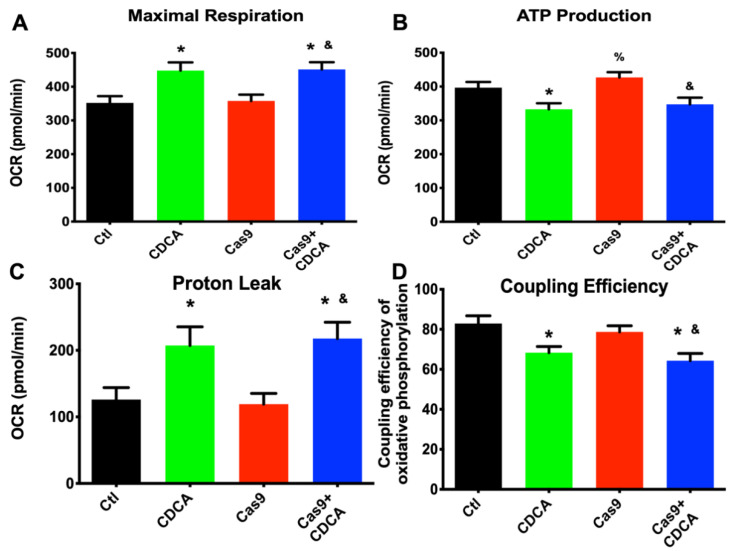
Seahorse respiration data obtained from 3T3-L1 adipocytes exposed to CDCA in the absence or presence of TGR5. (**A**) Maximal respiration; (**B**) ATP production; (**C**) Proton leak; (**D**) Oxidative phosphorylation coupling efficiency. Data are derived from 12 independent experiments, and bars represent means ± SEM. * indicates a statistically significant difference vs. Control (*p* < 0.05); % indicates a statistically significant difference vs. CDCA (*p* < 0.05); & indicates a statistically significant difference vs. Cas9 (*p* < 0.05).

Regarding the data obtained from a Seahorse extracellular fluxes analyzer, no differences in several oxygen consumption-related parameters were found, such as basal respiration, non-mitochondrial oxygen consumption, and spare respiratory capacity (data not shown). However, in the remaining ones (maximal respiration, ATP production, proton leak and coupling efficiency) there were significant alterations that the removal of TGR5 did not affect. As can be seen in Figure 2, CDCA affected mitochondrial efficiency of 3T3-L1 adipocytes independently of the TGR5 presence. In fact, CDCA boosted the maximal mitochondrial respiratory capacity, which was accompanied by an elevated protonic leak and diminished ATP production capacity, with a concomitant decreased coupling efficiency.

**Figure 3:**
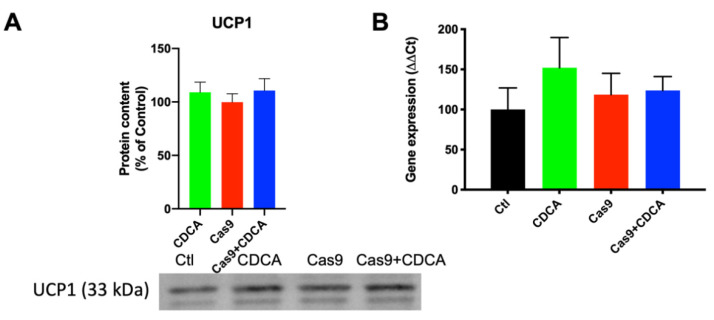
UCP1 levels in 3T3-L1 adipocytes exposed to CDCA, in the absence or presence of TGR5. (**A**) Representative Western Blot for UCP1 and respective quantification; (**B**) Gene expression for *ucp1*. Data are derived from seven independent experiments, and bars represent means ± SEM (no statistical differences were found).

Despite a trend for reduced acidification rate produced by the cells exposed to CDCA in regard to their respective control, no significant alterations in the ECAR (extracellular acidification rate) were found (data not shown). While it is true that elevated levels of lactate were found in non-CDCA conditions (as evaluated by NMR, Figure S1), since the cell media was refreshed prior to the assay, the lack of significant acidification is somewhat to be expected. Furthermore, the data in Figure S1 are the result of 96 h of metabolic activity, while the Seahorse ECAR measurement took place in a little over 2 h. As such, all this appears to indicate that CDCA is inducing ΔΨ consumption independently of ATP generation, which suggests that, here as well, is proof of an uncoupling effect. To assess if this was being mediated by the known mediator of BA activity in rodents, uncoupling protein 1 gene expression and protein content were next evaluated (Figure 3). Despite a trend towards elevated UCP1 expression and content in CDCA-exposed cells, no differences were found between any of the tested conditions.

**Figure 4:**
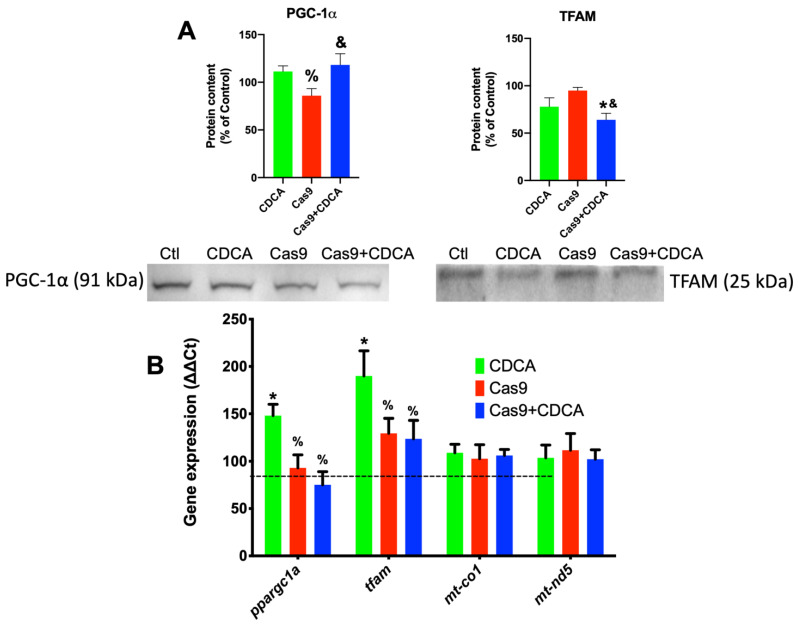
Gene expression and protein content for key elements of mitochondrial biogenesis in 3T3-L1 adipocytes exposed to CDCA in the absence or presence of TGR5. (**A**) Representative Western Blot for PGC-1α and TFAM; (**B**) Gene expression for PGC-1α (*ppargc1a*), TFAM (*tfam*), COX I (*mt-co1*), and mtND5 (*mt-nd5*). PGC-1a—Peroxisome proliferator-activated receptor gamma, coactivator-1alpha; TFAM—Transcription factor A, mitochondrial; COX I—Cytochrome c oxidase, subunit 1; mtND5—NADH-ubiquinone oxidoreductase, subunit 5. Data are derived from seven independent experiments, and bars represent means ± SEM. * indicates a statistically significant difference vs. Control (*p* < 0.05); % indicates a statistically significant difference vs. CDCA (*p* < 0.05); & indicates a statistically significant difference vs. Cas9 (*p* < 0.05).

As such, another explanation for these metabolic data could be alterations in mitochondrial content promoted by CDCA. To assess this, gene expression and protein levels of two key players of mitochondrial biogenesis (PGC-1α and TFAM) were assessed, as well as two genes coding for respiratory chain proteins, mtND5 and COX I. The results are show in Figure 4. It is apparent that CDCA tends to elevate the expression of both the genes coding for PGC-1α and TFAM (*ppargc1a* and *tfam*, respectively), an effect that is apparently lost when TGR5 is reduced (Figure 4B). However, in terms of protein content, while PGC-1α follows, for the most part, the gene expression pattern, TFAM has a total reversion of the pattern followed by the gene expression, with significant reduction on the Cas9 + CDCA group and a virtual (*p* = 0.0541) significance for the CDCA group, both when compared with their respective controls (Figure 4A). As such, it is difficult to fully understand the role CDCA on mitochondrial biogenesis given these data. However, no alterations regarding the expression of two mitochondrially-encoded respiratory chain genes (cytochrome *c* oxidase I and NADH-ubiquinone oxidoreductase chain 5, *mt-co1* and *mt-nd5*, respectively) were found, which might indicate that there is perhaps no significant effect of CDCA on mitochondrial biogenesis, for the tested conditions, at least on this experimental time frame. Regardless of this finding, it is safe to assume that if indeed CDCA is causing a loss of mitochondrial membrane potential, this will undoubtedly be sensed by the cell and will lead to the activation of the mitochondrial biogenic program, in order to prevent ATP generation losses. As such, these apparent contradictory data sets might be prey of the selected experimental time points.

### 2.4. CDCA Exposure Leads to Alterations in the Expression of Metabolically Relevant Players’ Genes

Since it appears that CDCA has dramatic effects on metabolism, both in vivo and in vitro, these effects in this cell model were further explored. As such, the gene expression of relevant agents in metabolic pathways was evaluated. Figure S3 illustrates the results obtained for the most relevant players tested. Here, it becomes clear that some gene expression pattern changes caused by CDCA were dependent on TGR5 presence (the genes for leptin and fatty acid binding protein 4, FABP4—also known as ap2), which indicates that TGR5 does indeed contribute to the metabolic alterations induced by bile acids. However, some other genes’ expression altered by CDCA exposure did not require normal levels of TGR5 (such as the ones coding for lipoprotein lipase, LPL or the peroxisome proliferator-induced receptor gamma, PPARγ), but for most of the genes evaluated, no significant alterations were found. As such, there is an inclination to conclude that if CDCA is indeed causing an acceleration of metabolic function in adipocytes in a mostly TGR5-independent fashion, without a concomitant alteration in key metabolic players, while at the same time altering mitochondrial biogenetic patterns, then CDCA might be promoting the improvement of metabolic function by the removal of incompetent/damaged mitochondrial units and promoting their replacement by newer, more efficient ones. These novel mitochondria could be the driving force behind the acceleration of metabolism and thus explain the results.

This conclusion is loosely based in the mitohormetic theory [16] which postulates that small injurious events to mitochondria lead not to dysfunction and damage to the cell, but the signaling of mitochondria for removal and degradation in an autophagic process (mitophagy) and their replacement with newer, non-damaged ones, resulting in a permanent population of highly effective and capable mitochondria, thus ensuing decreased damage and cell death. Of course, when the said injurious phenomena are too aggressive or prolonged, they do indeed lead to cellular dysfunction, but given the right agents and time frames, one could manipulate this cycle of mitochondrial degradation/renewal to force the cell to expend nutrients and thus explain the metabolic improvement results reported here. Since strong indications that CDCA leads to an increase in biogenic signals are present, it was thus sought to understand if the other side of the coin, the mitophagic process, was also indeed altered by CDCA exposure.

### 2.5. CDCA Leads to an Increase in Mitophagy Independently of TGR5’s Levels

CDCA’s effects on mitophagic fluxes were evaluated by quantifying the fluorescence of a novel mitophagy reporter. This fluorescent probe (Mtphagy dye) is a far-red fluorescent probe that selectively binds to mitochondria and gains an enormous boost in fluorescent intensity as it is exposed to very low pH (which, inside a living cell, tends to only occur within lysosomes and autophagosomes). To further verify if the fluorescence that was recorded was indeed caused by mitophagy, cells were also loaded with the green fluorescent Lysotracker Green probe, which selectively labels lysosomes. By combining the signals of both probes and only looking for overlapping of probe signals within a single adipocyte, enabling to accurately quantify mitophagic fluxes induced by CDCA, and how the knockdown of TGR5 would affect said fluxes. Furthermore, the gene expression and protein content of various players in mitochondrial dynamics which are heavily involved in the mitophagic process were also evaluated.

**Figure 5:**
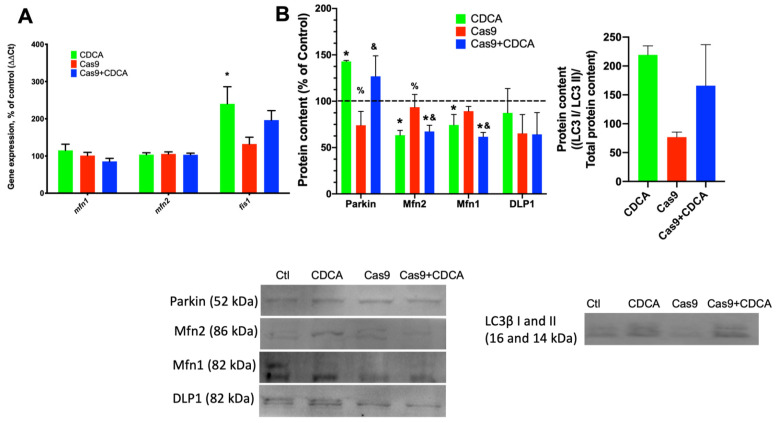
Mitophagy evaluation on 3T3-L1 adipocytes exposed to CDCA in the absence or presence of TGR5. (**A**) Gene expression of mitodynamics-involved proteins mitofusins 1 and 2 (*mfn1* and *mfn2*) and fission protein 1 (*fis1*); (**B**) Protein content of several players involved in mitophagy and mitochondrial fission and fusion. DLP1—Dynamin-like protein 1; Mfn1—Mitofusin 1; LC3—Light chain 3B; Mfn2—Mitofusin 2. Data are derived from 3 independent experiments, and bars represent means ± SEM. * indicates a statistically significant difference vs. Control (*p* < 0.05); % indicates a statistically significant difference vs. CDCA (*p* < 0.05); & indicates a statistically significant difference vs. Cas9 (*p* < 0.05).

As can be seen in Figure 5A, CDCA lead to an increase in the gene expression of *fis1*, a mitochondrial fission-related gene, while not affecting the fusion-related genes mitofusin 1 and 2 (*mfn1* and *mfn2*, respectively). Furthermore, protein content of these mitofusins is significantly decreased by CDCA in a TGR5-independent fashion, while simultaneously elevating parkin levels, a protein heavily involved in mitophagy (Figure 5B). Interestingly, despite a clear trend towards elevation caused by CDCA, there was no significant alteration to the protein levels of dynamin-like protein 1 (DLP1) and the microtubule-associated protein 1A/1B-light chain 3 (LC3), two other proteins involved in autophagy and mitophagy. These data suggest that mitochondrial fusion is decreased, while fission is increased, which is both a hallmark of elevated metabolic rates and indicative of increased mitophagic fluxes [16].

**Figure 6:**
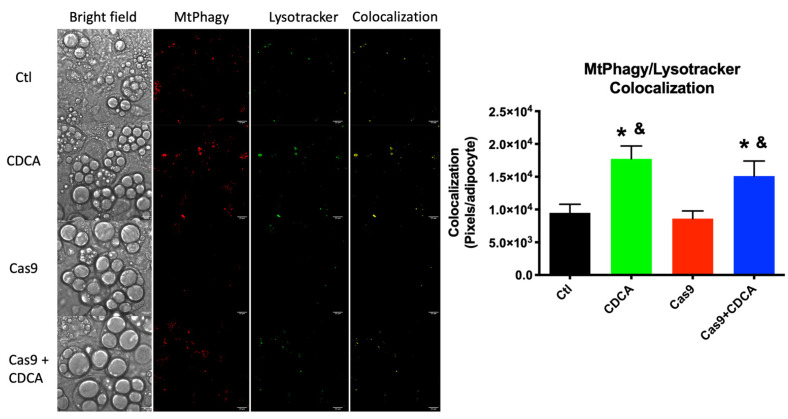
Mitophagy evaluation on 3T3-L1 adipocytes exposed to CDCA in the absence or presence of TGR5. Fluorescence microscopy representative images of the various experimental conditions, when observed under white light or evaluating fluorescent signal of the two probes, MtPhagy and Lysotracker Green. A fourth panel indicates the fluorescence signals’ colocalization where it happens, where it is represented in yellow. Scale bars in microscopy photographs represent a distance of 22 µm. Microscopy figures contrast settings were all maxed solely to gain visual clarity and long after image analysis. Bars represent means ± SEM of at least 185 individually quantified adipocytes. * indicates a statistically significant difference vs. Control (*p* < 0.05); & indicates a statistically significant difference vs. Cas9 (*p* < 0.05).

As such, the rates of mitophagy as reported by the fluorescent dyes MtPhagy and Lysotracker were assessed. Figure 6 shows that there was a dramatic increase in mitophagic events in both conditions in which CDCA was present, when compared with the control situations. As such, we can safely assume that CDCA (as probably other BA as well) not only leads to increase thermogenic dissipation in brown adipocytes, but also cause a mild uncoupling phenomenon which results in the removal of damaged mitochondria, dissipation of excess energetic potential, and thus reduction of nutrient overload and amelioration of the metabolic profile.

## 3. Discussion

Our previous work [3] sought to understand if the BA CDCA could also have metabolic effects on cultured 3T3-L1 white adipocytes, which was indeed the case. CDCA reversed many of the metabolic alterations induced by obesity in these cells, resulting in an acceleration of metabolic activity and mitochondrial function, despite the lack of a thyroid gland to supply the cells with T4 or ß-adrenergic stimulation. This led us to push further into trying to finally understand if the effects of BA on metabolism are solely driven by TGR5 on BAT or if there are other avenues of effect that are masked by the high relevance of BAT for small animals. To achieve this goal, the experimental design was intentionally driven away from a BAT model and more into the effects of the BA CDCA in a TGR5 CRISPR/Cas9 knockdown model.

The results reported here confirm that CDCA is capable of driving a metabolic acceleration and nutrient consumption in 3T3-L1 adipocytes despite the reduction in *gpbar1* and *dio2* expression (Figure 1). Triglyceride deposition analysis and metabolic profiling by NMR spectroscopy clearly indicates that CDCA does not require TGR5 for metabolic normalization (Figure S1), in a stark parallel to a previous work [3] and to the animal models [4,5,17]. Given this finding, the metabolic and mitochondrial effects of CDCA on these cells were further explored and what (if any) effects would the TGR5 reduced content cause. For the most part, this reduction did not affect the evaluated parameters. In fact, CDCA was TGR5-independently capable of reducing mitochondrial membrane potential and cellular ROS generation (Figure S2) and drive up the mitochondrial oxygen consumption by a clear reduction in membrane potential (Figure 2).

The next step was to understand if CDCA was inducing UCP1 levels as happens in the mice models used in previous studies. Encouragingly, UCP1 content was not altered, nor was its gene expression induced (Figure 3). This is in clear contrast with the animal data from virtually all previous studies and clearly indicated that there is probably another mechanism of mitochondrial membrane potential dissipation by CDCA that was being overlooked by the overbearing prevalence of thermogenic effects of BAT in mice. Interestingly, a recent work using a different BA (obeticholic acid, OBA), resulted in a UCP1 induction in vitro [18], which appears contradictory with our results. However, the cell line used in this study is a fibroblast cell line, functionally akin to a mesenchymal stem cell. As such, it is quite probable that OBA is inducing a brown fat differentiation program on these cells, a possibility clearly stated by the authors of that study [18].

Surprisingly, TGR5 ablation did have some effects on the mitochondrial biogenetic pathway, for CDCA was competent to induce the expression of both key players of mitochondrial biogenesis, *tfam* and *ppargc1a* (Figure 4), an effect that was lost in the Cas9 + CDCA cells. This indicates that TGR5 is intimately associated with mitochondrial biogenesis in BA-exposed cells, an effect that was virtually unknown until very recently [19]. Concomitantly, the expression of some metabolically-relevant genes was also affected by the reduction in TGR5 content, namely *lep* and *fabp4*, effects that were to be somewhat expected given the literature (respectively, [20,21]). Regardless, the expression of other genes relevant to metabolic function of adipocytes (*lpl* and *pparg*) were not affected by TGR5 reduction (Figure S3). Counter-intuitively, the expression of mitochondrial OXPHOS elements *mt-co1* and *mt-nd5* was also unaffected, either by CDCA or the reduction in TGR5, despite the increase in the expression of *tfam* and *ppargc1a* (Figure 4), which might indicate that the mitochondrial biogenetic protocol is not activated in these conditions, which makes some sense, since the activation of the oxidative phosphorylation illustrated in Figure S1, Figure S2 and Figure 2 is probably not sufficient to drive ATP levels down towards dangerous levels. Regardless of this finding, there are clear effects on the mitochondrial membrane integrity (illustrated by the reduction in membrane potential, ROS generation, and increased protonic leak) and, as such, it was thought that this might drive mitophagy up.

The mitophagic process is a series of subcellular events that lead to the labelling of damaged/inefficient/unnecessary mitochondrial units for degradation by autophagic means [22]. The mitodynamics involved revolve upon the decrease in mitochondrial fusion processes and elevated mitochondrial fission ones, with the unwanted fissioned fragment marked for degradation, typically by means of activation of the Parkin pathway [23]. As such, if indeed CDCA is causing a mild damage to mitochondria, this will certainly lead to an increase in mitophagic fluxes. We first investigated the gene expression and protein content of common players in mitodynamics and mitophagy (Figure 5A,B). Mitochondrial fusion proteins 1 and 2 gene expression was unaltered in all experimental conditions, while the gene for fission protein 1 (*fis1*) was elevated by CDCA. Concomitantly, the protein levels of Parkin were also found to be elevated by CDCA, independently of TGR5 levels. As such, the data highly suggests that CDCA is indeed causing an increase in mitophagy, which was confirmed by confocal microscopy analysis of data supplied by the use of a novel mitophagy fluorescent reporter [24]. The data unquestionably show that there is a virtual doubling of colocalization signal from the mitophagy and lysosome dyes within individual adipocytes, an effect that is independent of TGR5 levels (Figure 6). Since it has been known for some time that higher doses of BA lead to mitochondrial membrane potential dissipation and mitochondrial dysfunction [15], this work proposes that the explanation for the metabolic improvement seen in this model hinges not on thermogenic dissipation through the induction of UCP1 activity, but rather a very mild, survivable, dissipation of mitochondrial membrane potential that leads to a quasi mitohormetic effect, contributing to the improvement of the mitochondrial population efficiency and capacity [16], while, at the same time, removing excess nutrients in the form of elevated membrane potential, which has been known to be a major driver of obesity-related metabolic impairment [25]. While it is true that CDCA is not the most potent natural TGR5 agonist, the fact that FXR specific agonism has been shown to be harmful in an obesity setting explains this apparent limitation and justifies its use to, once and for all, try to explain these effects. In fact, by utilizing CDCA, this work highlights the fact that some very important effects of CDCA are not receptor dependent, as demonstrated by the data.

As for the strengths and weaknesses of this work, they mostly revolve around the experimental model. The fact that rodents, the commonly used experimental model in biomedical research, have such a potent and elevated dependency in BAT thermogenesis, any effect of BA that do not hinge on BAT activation by TGR5 agonism become incredibly difficult to correctly evaluate. As such, by using an in vitro model, molecular mechanisms and pathways that were overshadowed by BAT activation can thus be brought up for evaluation and measurement. Obviously, this work should be replicated in less BAT activation-prone models, as it would most interestingly be to evaluate bile acids effects in obese humans. Furthermore, it is tempting to assume that this phenomenon reported here is independent of target tissue, but given the intricate particularities of mitochondrial function and dynamics in the various tissues throughout the body, this question should be addressed in other cellular lines or, even more interestingly, in TGR5-KO animals. Finally, it would be important to also analyze if inhibition of mitophagy/autophagy with resource to chemical/pharmacological agents (for example, using Wortmannin) would indeed prevent the beneficial effects of BA.

In conclusion, this work proposes that BA have a yet unrecognized effect on mitochondria, achieving similar results to the current model of TGR5 activation, but mechanistically very different. This is extremely interesting for human applications, since BAT presence and activity in adult humans is still a matter of heated debate. The data suggests that CDCA might be a potent metabolic regulator in adult humans as it undoubtedly is in rodents.

## 4. Materials and Methods

Except when noted, all chemical reagents were purchased from Sigma-Aldrich (St. Louis, MO, USA) and were of the highest degree of purity commercially available.

### 4.1. Experimental Design for Cell Culture and TGR5 Silencing by CRISPR/Cas9

3T3-L1 fibroblasts (mycoplasm-free as evaluated by DAPI staining) were purchased from the American Type Culture Collection (ATCC, Manassas, VA, USA), and culturing and differentiation protocols were performed as before [3]. Briefly, cells were grown in 75-cm^2^ flasks (Sarstedt, Nümbrecht, Germany) with 15 mL Dulbecco’s modified eagle’s medium, DMEM (Sigma-Aldrich) 25 mM glucose, supplemented with 10% new-born calf serum, NCS (Thermo-Fisher, Waltham, MA, USA) and 1% penicillin–streptomycin mix (Thermo-Fisher) to sub-confluence, with routine medium changes every 48 h. Cells were subpassaged by trypsin-ethylenediaminetetraacetic acid, EDTA (Thermo-Fisher) detachment when confluence approached 80–90%.

Given the known resilience of mature 3T3-L1 adipocytes to manipulation and modification, which was confirmed by achieving very low silencing rates with silencing RNA (siRNA) delivered by both lipofectamine 2000, lipofectamine 3000, lipofectamine iMax, and electroporation with a Neon electroporator (all from Thermo-Fisher), it was decided to use an elegant protocol using Dharmacon’s Edit-R inducible CRISPR/Cas9 lentivirus (Horizon Discovery, Waterbeach, UK). These viruses contain a plasmid that codes for an inducible Cas9 endonuclease, which is transduced in the presence of doxycycline. Furthermore, the plasmid containing the Cas9 confers resistance to puromycin as a selection feature.

Fifty thousand cells were plated in 6-well multiwell plates (Sarstedt) in the same medium as above and, after 24 h, were exposed to the lentivirus containing the Cas9, with a multiplicity of infection (MOI) of 2. After 48 h, the medium was exchanged and 1 µM puromycin was added to each well, which resulted in the death of all non- or inefficiently-transfected cells. After 48 h, the medium was removed and normal DMEM + NCS were plated until the cell population had recovered. At this point, a second infection was performed (once more with a MOI of 2) with a sgRNA-coding plasmid contained in lentivirus (also from Dhamacon, Lafayette, CO, USA) that would guide the Cas9 towards the TGR5 gene (source clone ID: VSGMMM_27032603; Genomic location: mm10|-chr1:74279345–7429367; DNA target sequence: TAG TGG TGG GCG ACG CTC AT). This second lentiviral plasmid also conferred resistance to an antibiotic, in this case blasticidin; 1 µM blasticidin was thus used for a second cell selection. After the cell population had recovered its numbers, cells were ready for differentiation.

Subsequently, 50,000 cells were plated into each well of a 12-well multiwell plate in the same medium as above (DMEM + NCS). Upon achieving confluence, 48 h were then counted to achieve day 0 of differentiation, when the medium was exchanged for DMEM, as above, but with 10% fetal bovine serum instead of NCS, and also supplemented with 1 µg/mL insulin, 0.5 mM 3-isobutylmethyl-1-xantine (IBMX), 0.25 µM dexamethasone (DEX) and 2 µM rosiglitazone (ROSI). This medium was kept in the well for 48 h and then exchanged by the same medium, but without IBMX, DEX, and ROSI. Medium with FBS and Insulin was kept for the duration of the differentiation protocol, exchanged every 48 h until the differentiation was fully achieved (typically, over 70–80% of the cells achieve the morphology of adipocytes).

At this time point, Cas9 activity was induced by 1 µM doxycycline exposure for 96 h, after which the cells were exposed to 50 µM CDCA (or vehicle, DMSO), as before [3], still in the presence/absence of doxycycline. A full schematic of this protocol can be found in the supplementary materials (Figure S4). All assays were performed with cells at this point in differentiation and eventual Cas9 activation.

### 4.2. Intracellular Triglyceride Quantification

Adipocytical intracellular triglyceride deposition was quantified by Oil-Red O staining, as before [3]. Briefly, after treatments, cells were washed twice with warm phosphate-buffered saline buffer (PBS) and fixed by 30 min exposure to 4% paraformaldehyde in PBS, in an orbital shaking table. Cells were then washed twice more with PBS and once with distilled water, after which an overnight drying phase occurred. Plates were then incubated with Oil-Red O stain (6:4 Oil Red O stain 0.6% in isopropanol) for 1 h in the same orbital shaking table and washed three times with distilled water. Incorporated dye was dissolved in isopropanol, transferred into new multiwell plates and absorbance was recorded at 490 nm in a Victor 3 plate reader (Perkin-Elmer, Waltham, MA, USA).

### 4.3. Mitochondrial Membrane Potential (ΔΨ) and Reactive Oxygen Species (ROS) Evaluation

Mitochondrial membrane potential (ΔΨ) was measured by the use of a fluorescent dye, tetramethylrhodamine methyl ester (TMRM). TMRM is a cell-permeant cationic dye that is rapidly sequestered by living, respiring mitochondria due to the intramitochondrial negative charge accumulation. By working with a quenching concentration of TMRM of 6.6 µM, the mitochondrial matrix becomes overloaded with dye molecules, which effectively inhibit their own fluorescence by proximity quenching [26]. TMRM fluorescence was recorded at 590 nm after 485 nm excitation in the same plate reader as above, after a 15 min incubation with the probe and two washes with warm PBS to remove unloaded dye. After 10 min of basal recording of fluorescence, cells were exposed to a powerful mitochondrial uncoupler, 2 µM dinitrophenol (DNP). DNP destroys the ΔΨ and this leads to the release of trapped TMRM, thus removing the quenching block of fluorescence. Since TMRM molecules accumulate as a direct function of ΔΨ, the higher the fluorescence increase from basal to uncoupled is a direct indicator of how much ΔΨ the cell’s mitochondria possessed.

For reactive oxygen species generation (ROS) quantification, cells were loaded with 2′,7′-dichlorodihydrofluorescein diacetate (DCF-DA). DCF-DA is a cell-permeant dye that is quickly processed by intracellular enzymes, trapping and activating the probe within the cell, where it can contact hydrogen peroxide (H_2_O_2_) and thus become fluorescent [27]. Cells were loaded with 50 µM DCF-DA and incubated for 30 min, after which cells were washed twice with warm PBS and fluorescence was recorded in the same plate reader as above, with an excitation and emission wavelengths of 485 and 535 nm, respectively. Fluorescence was recorded for 10 min to establish a rate of ROS generation.

### 4.4. Mitochondrial Respiration Measurement

Mitochondrial respiration was evaluated by Seahorse XFe24 extracellular flux analysis (Agilent Technologies, Santa Clara, CA, USA). Briefly, at the start of the differentiation protocol, cells were plated in a Seahorse XFe24 plate instead of a 12-well multiwell plate, at 10,000 cells per well. All other differentiation and Cas9 induction steps were performed as above for the 12-well plates. On the day of the assay, cells were washed twice and incubated for 1 h with serum-free DMEM, as indicated by the manufacturer. The contents of the cell mito stress kit (Oligomycin, FCCP, Antimycin A/Rotenone mix, 1, 2, 0.5 µM final concentration respectively) were diluted in serum-free DMEM and loaded in a previously hydrated Seahorse sensor cartridge. After incubation, the cartridge and cell plate were loaded into the analyzer and a standard cell mito stress test protocol was run. Results were normalized by sulforhodamine B (SRB) quantification of cellular protein present in each well [3] and were analyzed using the Wave software (version 2.4.2) associated with the Seahorse equipment, also from Agilent.

### 4.5. Real-Time PCR

Gene expression analysis was performed by qPCR. RNA was extracted and purified by the Trizol method [28]. RNA was quantified with a Nanodrop One (Thermo-Fisher) and 500 ng of RNA were, according to manufacturer’s instructions, reverse transcribed with recourse to the iScript cDNA Synthesis kit (Bio-Rad, Hercules, CA, USA) in a Bio-Rad MyCycler. cDNA was diluted 1:10 and used for qPCR (performed in a Bio-Rad Mini-Opticon real-time PCR) with the use of the SsoAdvanced Universal SYBR Green Supermix (Bio-Rad). Primers used are listed in the supplementary methods section (Table S1). Data were normalized by the 2^-ΔΔCt^ method [29].

### 4.6. Western Blotting

The protein content in the cells was quantified by Western Blotting [30]. Briefly, after treatments, cells were washed twice with PBS and scrapped in Trizol [31]. Purified protein extracts were quantified by the bicinchoninic acid method and 50 µg of protein in Laemmli buffer were electrophoresed in lab-made sodium dodecyl sulphate-polyacrylamide gel electrophoresis (SDS-PAGE) gels, of the Criterion TGX type (Bio-Rad). This polyacrylamide has trihalo compounds embedded which bind to tryptophan residues in proteins, allowing the visualization of the protein in gel or after transfer. Gels were imaged using a GelDoc EZ (Bio-Rad). Separated proteins were then transferred to polyvinylidene difluoride (PVDF) membranes (Bio-Rad) via the use of a TransBlot Turbo apparatus (Bio-Rad). Membranes were blocked for 2 h in 5% bovine serum albumin (BSA) blocking buffer (Thermo-Fisher) and incubated overnight, at 4 °C with agitation, with primary antibodies (list of used antibodies can be found in the supplementary methods section, Table S2). The following morning, membranes were washed for 20 min twice in Tris-Buffered Saline Tween-20 0.1% (TBS-T) and incubated at room temperature with secondary antibodies for 1 h. After two more cycles of 20-min washes with TBS-T, membranes were protected from the light and incubated for 30 min with QDot 625 Streptavidin conjugate (Thermo-Fisher). Finally, membranes were imaged in a GelDoc EZ (Bio-Rad). Blots were normalized against total protein quantification of respective lanes from the same gel, as evaluated by imaging the gels, as previously mentioned, and following standard procedures [32,33]. Supplementary representative TGX gel images are supplied.

### 4.7. NMR Spectroscopy

Exposures and reagents used for NMR spectroscopy were as before [3]. ^1^H and ^13^C NMR spectra of aqueous extracts were acquired on a 14.1 Tesla Agilent VNMRS (Santa Clara, CA, USA) spectrometer using, respectively, a 3-mm indirect detection and a 3-mm broadband NMR probe. ^1^H NMR spectra consisted of 21 k points defining a spectral width of 7.2 kHz. A total of 64 scans was averaged to ensure adequate signal-to-noise ratios. In order to perform fully quantitative analysis typical acquisition parameters included a 30° radiofrequency observe pulse and an interpulse delay of 10 s to ensure full ^1^H relaxation. ^13^C NMR spectra consisted of 91 k points defining a spectral width of 37 kHz. Between 10k and 20k scans were averaged to ensure suitable signal-to-noise ratios for ^13^C multiplet quantification. Typical acquisition parameters included a 45° radiofrequency observe pulse and an interpulse delay of 3 s, to ensure full relaxation of aliphatic carbons. Before Fourier transformation, ^1^H free induction decays (FIDs) were zero filled and multiplied by a 0.2 Hz Lorentzian while for ^13^C FIDs, a 1 Hz Lorentzian was chosen. Spectral processing and deconvolution for quantitative analysis were performed using the NUTSpro™ NMR software (Acorn NMR Inc., Livermore, CA, USA).

### 4.8. Multivariate Analysis

Processed ^1^H NMR spectra were bucketed using all intensity values after excluding the water area and areas without any peaks (Amix Viewer, version 4.0.1, BrukerBiospin, Rheinstetten, Germany). Spectra were aligned using ico-shift algorithm [34] and normalized by total spectral area. Obtained matrix was subjected to multivariate analysis (MVA) including principal component analysis (PCA) and partial-least square discriminant analysis (PLS-DA) using SIMCA 14.1 (Umetrics, Sweden) [3]. PLS-DA models were validated by a 7-fold internal cross-validation and permutation test (*n* = 200). The PLS-DA loading plots were obtained by multiplying loading weights with responding standard deviations and were colour coded according to variable importance to the projection (VIP) using statistical software R (R Core Team). The quantitative assessment of group differences was performed by individual peak integration (Amix-Viewer, version 4.0.1, BrukerBiospin, Rheinstetten, Germany). Metabolite peaks contributing to the group separation (PLS-DA VIP > 1) were integrated, normalized, and the statistical difference was assessed by ANOVA followed by Sidak’s multiple comparison test.

### 4.9. Mitochondrial Mitophagy Evaluation

Mitophagic activity was evaluated with a kit and following the manufacturer’s recommendations (Mtphagy kit, Dojindo Molecular Technologies, Kumamoto, Japan). Briefly, 10,000 cells were plated in µ-slide 8-well plates (Ibidi GmbH, Gräfelfing, Germany) and differentiation and treatments occurred as described above, after which cells were washed twice with warm HEPES-buffered Hanks’s solution and incubated in this medium with 50 nM Mtphagy dye for 30 min. After this period, cells were once more washed twice with warm HEPES-buffered Hanks and incubated with 10 nM Lysotracker Green dye, for 30 min. Finally, cells were washed twice with HEPES-buffered Hanks and imaged by confocal fluorescence microscopy (Maker and model: Carl Zeiss LSM 710; Type, magnification and numerical aperture of the objective lens: Plan Apochromat 63x/1.4; Temperature: controlled room temperature of 20 °C; Medium: Hanks Balanced Salt Solution, (Sigma-Aldrich); Fluorochromes: Lysotracker Green and Mtphagy dye, both from Dojindo EU, Munich, Germany; Camera maker and model: Not applicable; Acquisition software: Zen Black, Carl Zeiss, Jena, Germany). Images were stacked in Image J software (NIH, Bethesda, MD, USA) and a built-in script that looked for overlapping of signal of both channels was run using the Pearson’s correlation coefficient [35]. Briefly, this method converts the images from both channels to B&W, where the fluorescent signal is marked as a white pixel. Thus, where white pixels overlap between the two figures, the software counts them as positive events. This is performed for each individual adipocyte in the photograph by hand-drawn limitation of the area of interest to the software. In total, for each experimental group, a *n* of 4 different experiments, each with 6 repeats, was counted, which amassed to at least 185 individual adipocytes analyzed per treatment.

### 4.10. Statistical Analysis

Statistical analysis was performed using Graphpad Prism 6 for Mac OS (San Diego, CA, USA). One-way ANOVA with Fisher LSD post-hoc tests were performed, and a *p* value of < 0.05 was deemed statistically significant. The sample size was not calculated by statistical methods, but was determined based on previous experience. Experimenters were not blinded to the group assignment, but were for the outcome assessment.

## Data Availability

All data are available upon reasonable request to the corresponding author.

## Supplementary Materials

The following are available online at https://www.mdpi.com/article/10.3390/ijms222111738/s1.
