# Supplementary material for: Chenodeoxycholic Acid Has Non-Thermogenic, Mitodynamic Anti-Obesity Effects in an In Vitro CRISPR/Cas9 Model of Bile Acid Receptor TGR5 Knockdown
Source: Int J Mol Sci. 2021 Oct 29;22(21):11738. doi: 10.3390/ijms222111738 (PMC8584144; doi:10.3390/ijms222111738)
Supplement: Supplementary file 1 [file ijms-22-11738-s001.zip › Supplementary Data.pdf]

Supplementary Materials

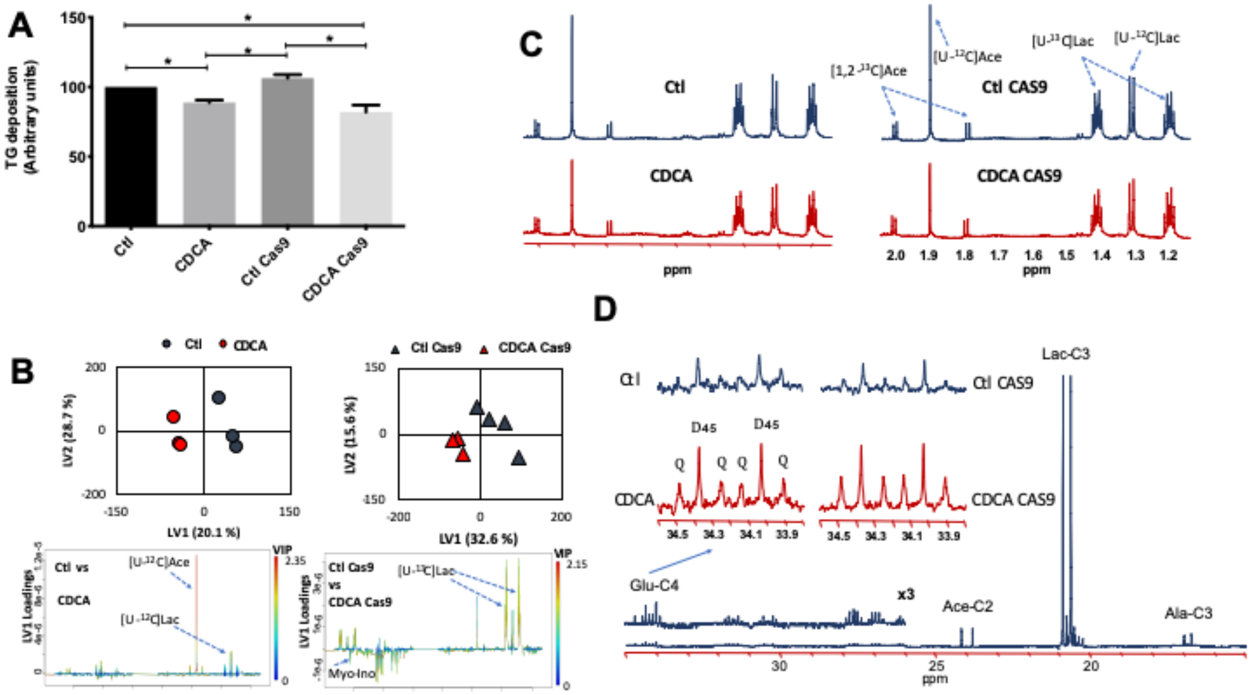

Figure S1 – Metabolic characterization of the effects of CDCA and dependency on TGR5. (A) Oil-Red O staining of triglyceride (TG) deposition; **(B)** PLS-DA scores scatter plots (above) and loadings plots (below) of Ctl versus CDCA (left) and Cas9 versus CDCA+Cas9 (right) of aqueous cell extracts. PLS-DA loadings are colored according to VIP values and some metabolite assignments are indicated: Ace: acetate, Lac: lactate, Myo-ino: myo-inositol; **(C)** Expansions (1.14-2.04 pp) of ^{1}H NMR spectra from cell culture media of Ctl, CDCA, Cas9 and CDCA+Cas9 cells. Levels of ^{13}C lactate ([U-^{13}C]Lac) are significantly higher in Ctl and Cas9 cells while the ^{13}C enrichment of acetate ([1,2-$^{13}C_2$]Ace) are higher in CDCA and CDCA+Cas9 cells; **(D)** Expansions of ^{13}C NMR spectra from the aqueous cell extracts of Ctl, CDCA, Cas9 and CDCA+Cas9 cells showing the multiplets of carbon 4 of glutamate (Glu-C4 (34.2 ppm)). The doublet 45 (D45) and pseudoquartet (Q) multiplets are significantly more intense in CDCA and CDCA+Cas9 cells than in the Ctl and Cas9 cells. Data are representative of 8 (A) or 6 (B-D) different independent experiments. For panel A, bars represent means ± SEM (* indicates a statistically significant difference within the bar).

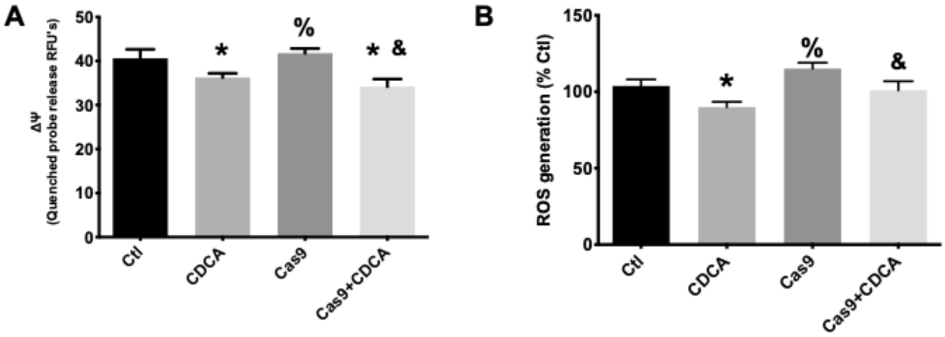

Figure S2 – Mitochondrial membrane potential and celular ROS generation in 3T3-L1 adipocytes exposed to CDCA in the absence or presence of TGR5. (A) TMRM fluorescence; **(B)** H_2DCF-DA fluorescence. Data are derived from 6 independent experiments, and bars represent means ± SEM (* indicates a statistically significant difference vs Ctl; %

indicates a statistically significant difference vs CDCA; & indicates a statistically significant difference vs Cas9. $p < 0.05$).

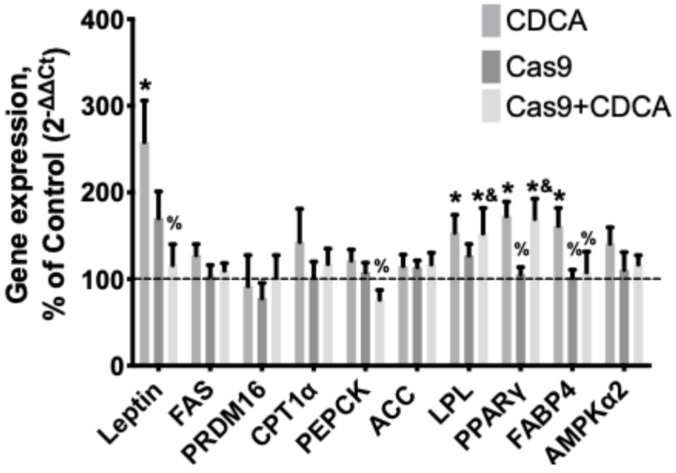

Figure S3 – Expression of several genes of 3T3-L1 adipocytes exposed to CDCA, in the presence or absence of TGR5. FAS – Fatty acid synthase; PRDM16 – PR domain containing 16; CTP*1α* – Carnitine palmitoyltransferase 1A; PEPCK – Phosphoenolpyruvate carboxykinase; ACC – Acetyl-CoA carboxylase; LPL – Lipoprotein lipase; PPARγ – Peroxisome proliferator-activated receptor gamma; FABP4 – Fatty acid-binding protein 4, a.k.a., adipocyte protein 2; AMPKα2 – 5'-AMP-activated protein kinase catalytic subunit alpha-2. Data are derived from at least 5 independent experiments, and bars represent means ± SEM (* indicates a statistically significant difference vs Ctl; % indicates a statistically significant difference vs CDCA; & indicates a statistically significant difference vs Cas9).
