# Supplementary material for: Chenodeoxycholic Acid Has Non-Thermogenic, Mitodynamic Anti-Obesity Effects in an In Vitro CRISPR/Cas9 Model of Bile Acid Receptor TGR5 Knockdown
Source: Int J Mol Sci. 2021 Oct 29;22(21):11738. doi: 10.3390/ijms222111738 (PMC8584144; doi:10.3390/ijms222111738)

Supplementary Figure WB – TGX total protein gels. MW, molecular weight marker; 1 – Ctl; 2 – CDCA; 3 – Cas9; 4 – Cas9 + CDCA.

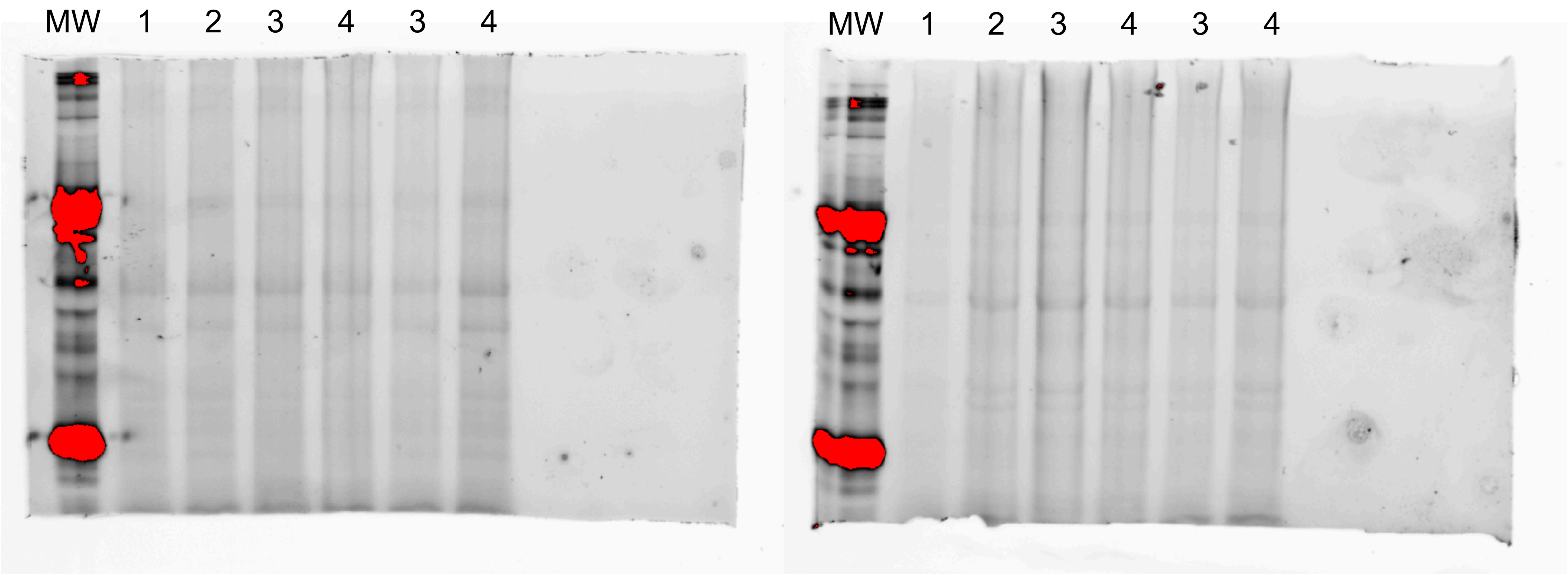

Supplement: Supplementary file 1 [file ijms-22-11738-s001.zip › Supplementary Figure WB.pdf]
