# Supplementary material for: Chenodeoxycholic Acid Has Non-Thermogenic, Mitodynamic Anti-Obesity Effects in an In Vitro CRISPR/Cas9 Model of Bile Acid Receptor TGR5 Knockdown
Source: Int J Mol Sci. 2021 Oct 29;22(21):11738. doi: 10.3390/ijms222111738 (PMC8584144; doi:10.3390/ijms222111738)
Supplement: Supplementary file 1 [file ijms-22-11738-s001.zip › Supplementary Schematic.pdf]

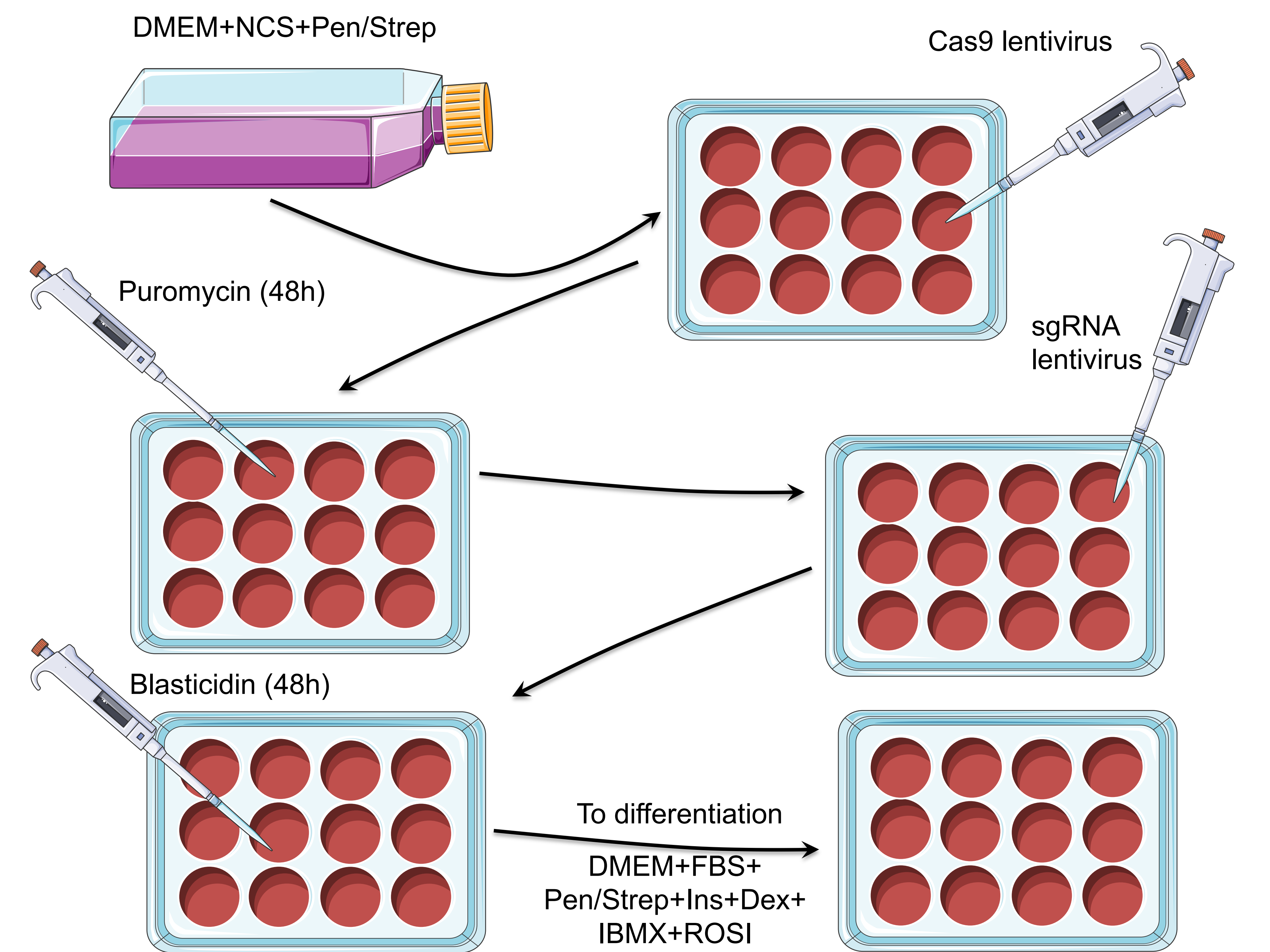
DMEM+NCS+Pen/Strep
Cas9 lentivirus
Puromycin (48h)
sgRNA lentivirus
Blasticidin (48h)
To differentiation
DMEM+FBS+ Pen/Strep+Ins+Dex+ IBMX+ROSI

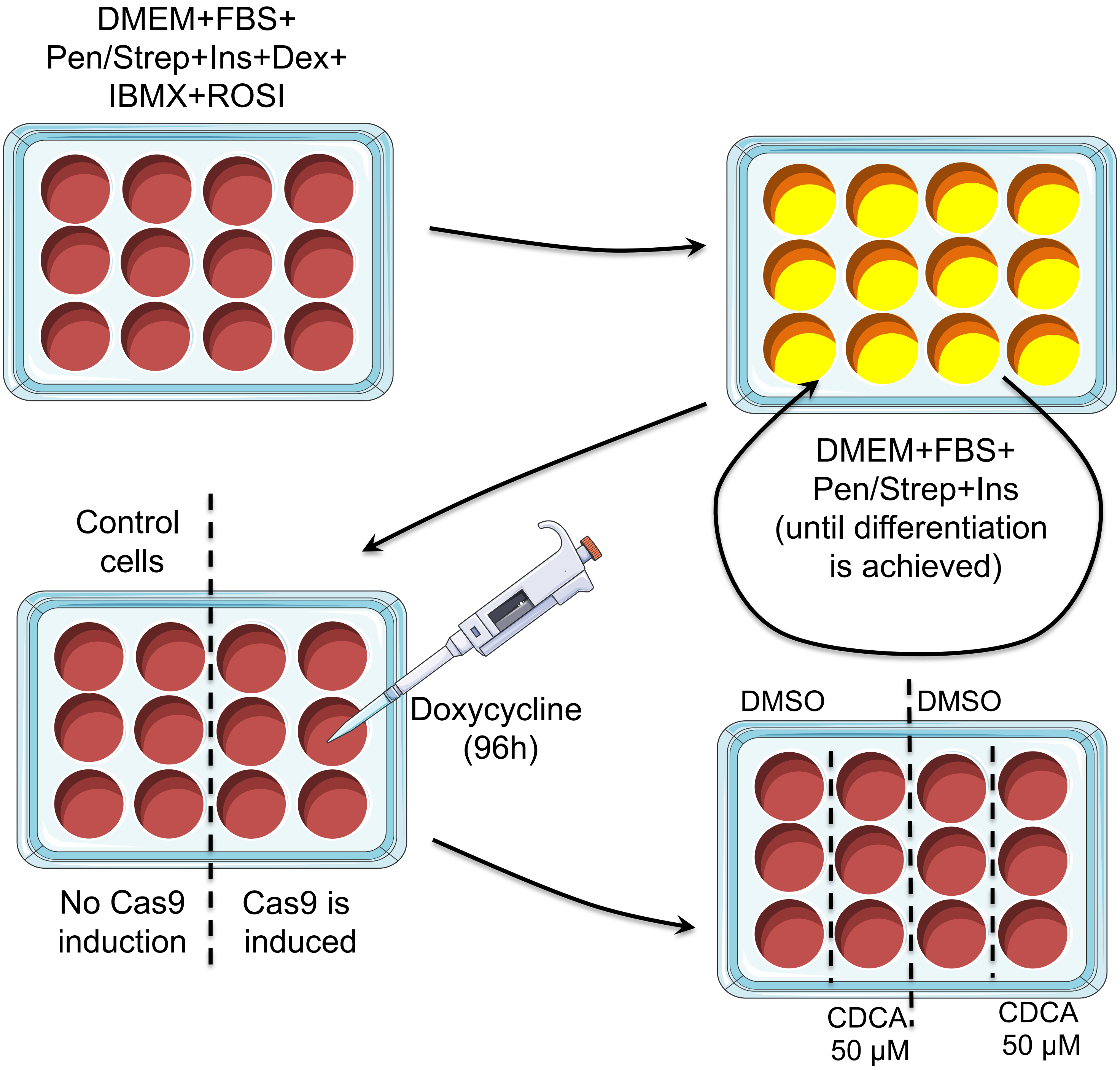
DMEM+FBS+
Pen/Strep+Ins+Dex+
IBMX+ROSI
DMEM+FBS+
Pen/Strep+Ins
(until differentiation
is achieved)
Control
cells
Doxycycline
(96h)
No Cas9
induction
Cas9 is
induced
DMSO
DMSO
CDCA
50 μM
CDCA
50 μM
