# Supplementary material for: Chenodeoxycholic Acid Has Non-Thermogenic, Mitodynamic Anti-Obesity Effects in an In Vitro CRISPR/Cas9 Model of Bile Acid Receptor TGR5 Knockdown
Source: Int J Mol Sci. 2021 Oct 29;22(21):11738. doi: 10.3390/ijms222111738 (PMC8584144; doi:10.3390/ijms222111738)
Supplement: Supplementary file 1 [file ijms-22-11738-s001.zip › Supplementary Table S1.pdf]

Supplementary Table S1 – qPCR primer sequences used.

Gene name	Sequence	NCBI's Nucleotide accession number
ACC1	TCC GCA CTG ACT GTA ACC AC; GTT TGT CAG GAA GAG GCG GA	NM_133360.2
AMPKα2	AGC AGC TGG GTT TTG AAT GGA AGG T; GGA GGC CCG CAG CAG AAC AGG	NM_178143.2
COX I	CCC AGA TAT AGC ATT CCC ACG; ACT GTT CAT CCT GTT CCT GC	NC_005089.1
CPT1α	GCA GCT CGC ACA TTA CAA GGA CAT; AGC CCCCGC CAC AGG ACA CAT AGT	NM_013495.2
D2	CCT CTT CCT GGC GCT CTA TG; TCA GGA TTG GAG ACG TGC AC	NM_010050.4
FABP4	TGA AAG AAG TGG GAG TGG GC; TTC ACC TTC CTG TCG TCT GC	NM_024406.3
FAS	GGC TGC CTC CGT GGA CCT TAT C; GTC TAG CCC TCC CGT ACA CTC ACT CGT	NM_007988.3
FIS1	AGA TGG ACT GGT AGG CAT GG; CTA CAG GGG TGC AGG AGA AA	NM_025562.3
FXR	CCC CTG CTT GAT GTG CTA CA; GTC CAT CAC TGC ACA TCC CA	NM_009108.2
LEPTIN	TTC GTG CTC AGC TCT GTC TG; GTC ACC CTC AGC TCA GGT TC	NM_008493.3
LPL	GGG CTC TGC CTG AGT TGT AG; CCA TCC TCA GTC CCA GAA AA	NM_008509.2
MITOFUSIN1	GCT GTC AGA GCC CAT CTT TC; CAG CCC ACT GTT TTC CAA AT	NM_024200.4
MITOFUSIN2	GCC AGC TTC CTT GAA GAC AC; GCA GAA CTT TGT CCC AGA GC	NM_001285920.1
ND5	TGG ATG ATG GTA CGG ACG AA; TGC GGT TAT AGA GGA TTG CTT GT	NC_005089.1
PEPCK	GCA GTG AGG AAG TTC GTG GA; GAT CCT GGC CAC ATC TCG AG	NM_011044.3
PGC-1α	CAC CAA ACC CAC AGA AAA CAG; GGG TCA GAG GAA GAG ATA AAG TTG	NM_008904.2
PPARγ	GGC GAG GGC GAT CTT GAC AGG; GAA ACT GGC ACC CTT GAA AA	NM_011146.3
PRDM16	AGG GCA AGA ACC ATT ACA CG; AGA GGT GGT CGT GGG TAC AG	NM_027504.3
SHP	TGT GTG AAG TCT TGG AGC CC; CAG ACT CCA TTC CAC GGG TC	NM_011850.3
TFAM	CTT TGA GCC TTG ACA GAA G; ATA TGT AAC GGT CAT CAG TG	NM_009360.4
TGR5	TGG AAG TTT ATG GCC TCC TG; CCA ACA CAG CAA GAA GAG CA	NM_174985.1
UCP1	CAC GGG GAC CTA CAA TGC TTA CAG; ACT GGA GAG GCC AGG AGT GT	NM_009463.3
