# Supplementary material for: Chenodeoxycholic Acid Has Non-Thermogenic, Mitodynamic Anti-Obesity Effects in an In Vitro CRISPR/Cas9 Model of Bile Acid Receptor TGR5 Knockdown
Source: Int J Mol Sci. 2021 Oct 29;22(21):11738. doi: 10.3390/ijms222111738 (PMC8584144; doi:10.3390/ijms222111738)
Supplement: Supplementary file 1 [file ijms-22-11738-s001.zip › Supplementary Table S2.pdf]

Supplementary Table S2 – Western Blotting antibodies used. Primary antibodies were used at 1:1000 dilution in TBS-T supplemented with 5% blocking reagent (Thermo-Fisher). Secondary antibodies were diluted 1:2500 in TBS-T.

Protein	Host species	Supplier	Catalogue #
DLP1	Mouse	BD Biosciences	611112
LC3	Rabbit	Sigma-Aldrich	L7543
Mfn1	Rabbit	Abcam	Ab104274
Mfn2	Rabbit	Abcam	Ab50838
Parkin	Rabbit	Cell Signaling Technology	2132
PGC-1α	Rabbit	Cell Signaling Technology	2178
TFAM	Rabbit	Aviva Systems Biology	ARP36993
UCP1	Rabbit	Abcam	Ab10983
Biotin XX, anti-mouse IgG	Goat	Thermo-Fisher	W10132
Biotin XX, anti-rabbit IgG	Goat	Thermo-Fisher	W10142
